# Divergent Selection and the Evolution of Signal Traits and Mating Preferences

**DOI:** 10.1371/journal.pbio.0030368

**Published:** 2005-10-25

**Authors:** Howard D Rundle, Stephen F Chenoweth, Paul Doughty, Mark W Blows

**Affiliations:** **1**School of Integrative Biology, University of Queensland, St. Lucia, Queensland, Australia; The University of North CarolinaUnited State of America

## Abstract

Mating preferences are common in natural populations, and their divergence among populations is considered an important source of reproductive isolation during speciation. Although mechanisms for the divergence of mating preferences have received substantial theoretical treatment, complementary experimental tests are lacking. We conducted a laboratory evolution experiment, using the fruit fly *Drosophila serrata,* to explore the role of divergent selection between environments in the evolution of female mating preferences. Replicate populations of *D. serrata* were derived from a common ancestor and propagated in one of three resource environments: two novel environments and the ancestral laboratory environment. Adaptation to both novel environments involved changes in cuticular hydrocarbons, traits that predict mating success in these populations. Furthermore, female mating preferences for these cuticular hydrocarbons also diverged among populations. A component of this divergence occurred among treatment environments, accounting for at least 17.4% of the among-population divergence in linear mating preferences and 17.2% of the among-population divergence in nonlinear mating preferences. The divergence of mating preferences in correlation with environment is consistent with the classic by-product model of speciation in which premating isolation evolves as a side effect of divergent selection adapting populations to their different environments.

## Abstract 2

Experimentally manipulating the resource environment of *Drosophila serrata* reveals that mating preferences can evolve, at least in part, as a result of environmentally-based divergent natural selection.

## Introduction

It is common in natural populations for individuals of one sex (usually females) to prefer certain trait values over others in their choice of mates [1–5]. Such mating preferences have long been thought to be key to speciation, because their divergence among populations will generate premating (behavioral) reproductive isolation. Consistent with this, mating preferences have been observed to vary among populations and closely related species in nature [5–10], and, in several taxa, evidence suggests that the resulting behavioral isolation has been instrumental in initiating speciation [11].

According to various speciation models, divergence among populations in mating preferences can occur in two main ways [12]. First, initial divergence in mating preferences in arbitrary directions may be caused by chance events such as unique mutations and/or the order in which they appear. Although such genetic drift is unlikely to cause substantial preference divergence on its own [11], sexual selection may subsequently amplify this initial divergence to yield a wide array of possible outcomes [13–16]. Speciation by sexual conflict is a popular example of such a model [16–18]. Second, initial divergence in mating preferences may be caused by divergent natural selection between environments, which may also in some [19–21], but not all [22] models, be subsequently amplified by sexual selection.

The roles of genetic drift and divergent natural selection in the diversification of mating preferences are not well understood. A number of comparative studies implicate sexual selection in speciation in a variety of taxa [11], although comparative approaches are unable to provide direct tests of the evolutionary mechanisms responsible for the initial divergence in mating preferences. The role of divergent natural selection in the evolution of premating isolation has been tested experimentally, with results clearly demonstrating the feasibility of this mechanism under some conditions [11,12,23]. Unfortunately, how divergent selection generates premating isolation is typically not known, because the mating preferences responsible are generally not identified in such experiments. A complementary experimental approach to understanding the mechanisms responsible for the diversification of mating preferences has not been developed. Such an approach is important, because the details of signal trait and preference evolution are key to distinguishing various speciation models [11].

Here we present an evolutionary experiment designed to directly test the role of divergent selection in the diversification of mating preferences. A clear expectation of how divergent selection should affect mating preferences is provided by the classic by-product model of allopatric speciation. According to this model, reproductive isolation evolves as a side effect of divergent selection adapting populations to their different environments [12,23–25]. If differences in mating preferences are a key trait contributing to reproductive isolation, independent populations adapted to different environments should diverge in mating preferences, whereas those adapted to similar environments should express the same preference. Laboratory experiments [23,26,27] and studies in nature [12,28–30] have confirmed these predictions for the evolution of premating isolation. Here, we provide an experimental test of whether these same predictions can be verified for the evolution of female mating preferences.

We tested how adaptation to two novel resource environments affected the evolution of female mating preferences in *Drosophila serrata,* a species in which mate choice has been investigated in a number of genetic and evolutionary experiments. *D. serrata* uses multiple contact pheromones, composed of nonvolatile cuticular hydrocarbons (CHCs), in both mate choice within populations [31–35] and species recognition [36,37]. Most importantly, male CHCs have been shown to respond rapidly to both natural and sexual selection [37,38], demonstrating that these signal traits readily evolve when selection is manipulated. However, how female mating preferences for male CHCs respond to divergent selection has not been determined.

We do so here by deriving 12 replicate populations from a common ancestor and propagating four of them in each of three separate treatment environments: their ancestral laboratory environment (yeast food) and two novel environments (rice and corn food). We show how the novel environments affected the evolution of CHCs and female mating preferences for them using a three-stage process. First, we demonstrate that CHCs adapted to the novel environments using the classic pattern of parallel evolution. Parallel evolution provides strong evidence that divergent selection between environments is responsible for trait evolution, because other mechanisms of evolution, such as genetic drift, are unlikely to produce similar changes in independent populations in correlation with environment [39,40].

Second, we demonstrate for each population the importance of CHCs in determining male mating success by employing population-level sexual selection gradients to estimate the form and strength of female mating preferences for male CHCs. Like many signaling systems [41,42], mate choice in *D. serrata* depends on the collective presence of multiple traits; here we consider the nine male CHCs shown by past studies to be associated with male mating success [31–33,35] and species recognition [36,37]. Third, to deal with the complexity generated by estimating 528 separate sexual selection gradients within a single experimental design, we employ a multivariate model fitting approach that uses partial *F*-tests to partition the effects of linear (directional) and nonlinear (quadratic and correlational) sexual selection within and among treatment environments. A role for divergent selection in preference evolution is demonstrated by consistent changes in preferences in correlation with treatment environment.

## Results

### Adaptation of Male and Female CHCs

**Figure 1:**
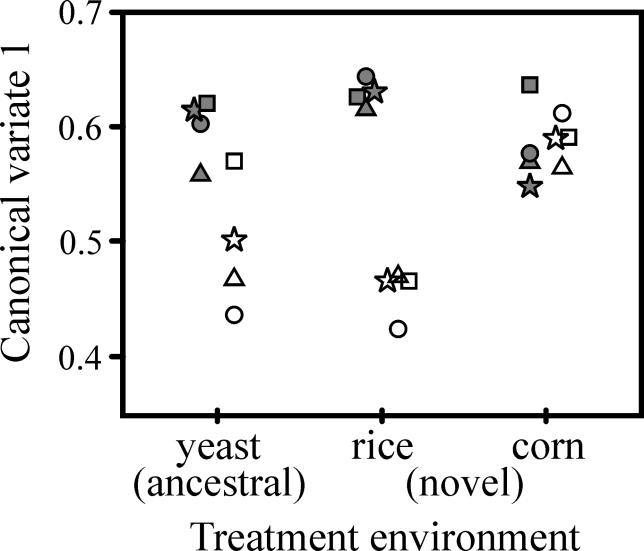
Adaptation of Male and Female CHCs to the Different Treatment Environments Variation among populations is presented as the first CV of the sex × treatment interaction from a MANOVA of the eight logcontrast CHCs of individuals from the 12 populations. Males are represented by filled symbols, and females by open symbols. The four replicate populations within each treatment are indicated by the different shaped symbols (there is no correspondence among treatment environments of populations represented by the same symbol).

CHCs adapted to the novel food environments, evolving in parallel in correlation with these environments. As indicated by the significant sex × treatment interaction (Table 1), the response to selection differed in males and females. CHCs also varied significantly among populations within the treatment environments (Table 1). Examination of the first canonical variate (CV) of the sex × treatment interaction (CV_1_, the linear combination of eight logcontrast-transformed CHCs that explains the most variance—85.2% in this case—in the sex × treatment interaction) reveals that, relative to the populations in the ancestral yeast environment, sexual dimorphism in the combination of CHCs that responded to selection tended to increase in populations adapted to rice and decrease in populations adapted to corn (Figure 1). When the sexes were analyzed separately, the treatment effect was significant in females (*p* = 0.018) but not in males (*p* = 0.153), indicating greater adaptation to the treatment environments by females than males.

**Table 1:**
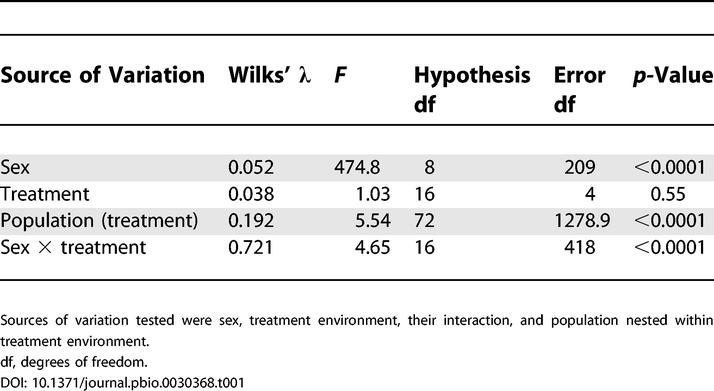
MANOVA Testing the Effects of Various Sources of Variation on the Eight Logcontrast Transformed CHCs Measured on Virgin Flies from 12 Experimental Populations of *D. serrata*

### Sexual Selection on Male CHCs Within Populations

Consistent with results from past studies [31–35], female mating preferences generated strong sexual selection on male CHCs in these populations (Table S1). Overall, linear sexual selection on the eight logcontrast CHCs was significant in each of the 12 populations (*p* < 0.0001 in all cases) and explained 29%–59% (mean 46%) of the variance in male mating success (Table 2). The addition of all nonlinear sexual selection was also highly significant overall in each of the 12 populations (*p* < 0.0001 in all cases), and the combination of linear and nonlinear selection explained 38%–68% (mean 56%) of the variance in male mating success (Table 2).

**Table 2:**
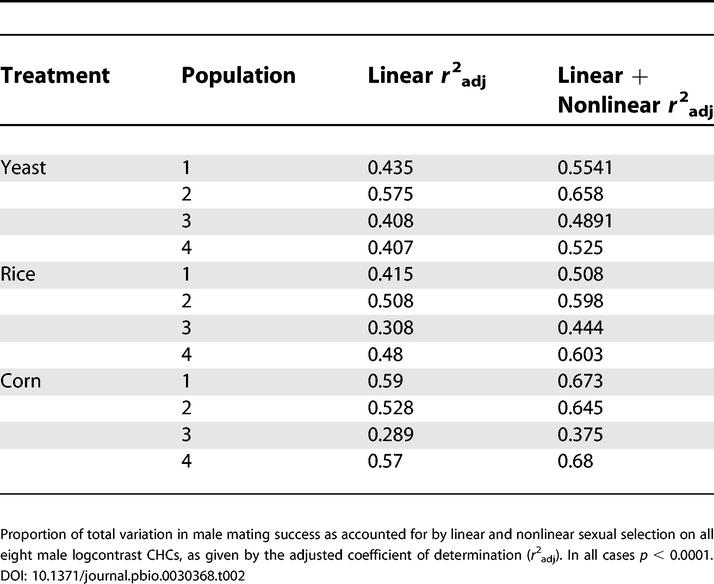
Proportion of Total Variation in Male Mating Success Accounted for by Sexual Selection on Male CHCs

### Variation among Populations and Treatments in Sexual Selection on Male CHCs

Sexual selection on male CHCs generated by female mating preferences varied significantly among populations overall, both for linear and nonlinear selection (partial *F*-tests, *p* < 0.0001 in both cases; Table 3). This indicated that female mating preferences diverged among populations.

**Table 3:**
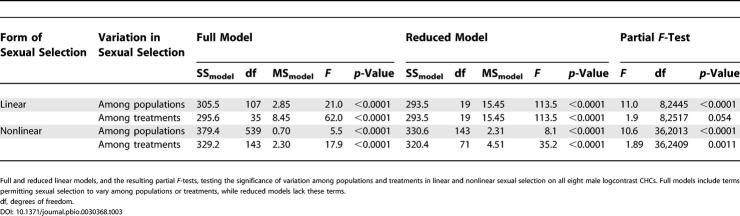
Linear Models and Partial *F*-Tests on Significance of Variation

Sexual selection on the eight male logcontrast CHCs also varied consistently among treatment environments, indicating that at least a component of the among-population divergence in mating preferences occurred in correlation with environment. This variation approached significance for linear selection (partial *F*-test, *p* = 0.054; Table 3) and accounted for at least 17.4% of the among-population variation in linear selection. For nonlinear sexual selection, this among-treatment variation was highly significant (partial *F*-test, *p* = 0.0011; Table 3) and accounted for at least 17.2% of the among-population variation in nonlinear selection. Because the overall importance of CHCs in explaining variation in male mating success was similar in all three environments (see Table 2), there is no indication that females in either novel environment became more or less choosy with regard to male CHCs. Rather, it is the combinations of male CHCs associated with mating success that changed.

**Figure 2:**
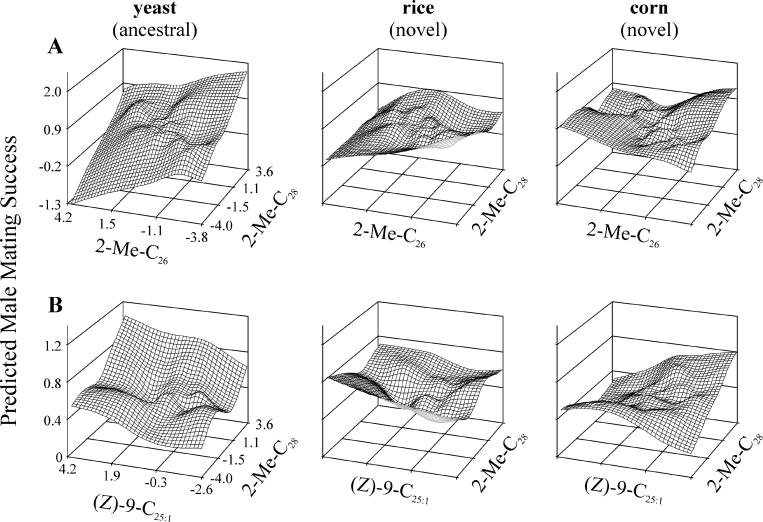
Thin-Plate Spline Representations of Bivariate Fitness Surfaces for Male CHCs for Which Female Mating Preferences Evolved in Correlation with Treatment Environment (A) Visualization of the fitness surface of the two male CHCs for which linear sexual selection varied most among treatments. (B) Visualization of the fitness surface of the two male CHCs for which nonlinear sexual selection varied most among treatments. The four replicate populations within each treatment were pooled in each case. To aid in comparisons across treatments, a single smoothing parameter (λ) was chosen that gave the lowest generalized cross-validation score in all three treatments [43] separately for (A) (λ = −1.0) and (B) (λ = −0.2).

Variation among treatments in linear sexual selection was greatest for CHC 2-Me-C_26_, although the univariate interaction of this trait with treatment was not significant (*F*_2,2517_ = 2.47, *p* = 0.085). The next greatest contribution to among-treatment variation in linear sexual selection was made by CHC 2-Me-C_28_, although again the univariate interaction of this trait with treatment was non-significant (*F*_2,2517_ = 1.55, *p* = 0.213). To provide a visual interpretation of how sexual selection varied among treatment environments, we used nonparametric thin-plate splines [43] to explore the bivariate fitness surfaces associated with these two CHCs. In the ancestral yeast environment, the fitness surface resembled a sloping plane, consistent with past experiments in this environment [33]. In the two novel environments, the slope of the fitness surfaces decreased, and combinations of CHCs that were unattractive in the ancestral environment (i.e., high values of 2-Me-C_26_ and low values of 2-Me-C_28_) appeared to have increased in attractiveness (Figure 2A).

Among-treatment variation in nonlinear sexual selection was contributed to most by correlational selection between CHCs (Z)-9-C_25:1_ × 2-Me-C_28_ and (Z)-9-C_25:1_ × 2-Me-C_26_; univariate trait × treatment interactions were significant for each ([Z]-9-C_25:1_ × 2-Me-C_28_, *F*_2,2409_ = 7.04, *p* < 0.001; [Z]-9-C_25:1_ × 2-Me-C_26_, *F*_2,2409_ = 4.41, *p* = 0.012). The nonparametric visualization of the bivariate fitness surface generated by the first pair of CHCs (Figure 2B) indicates how correlational selection between them has changed across treatments. The highest fitness combination in the ancestral yeast environment (i.e., high values of both [Z]-9-C_25:1_ and 2-Me-C_26_) has low mating success in the corn environment, changing the curvature along one diagonal of the surface from concave (bowl-shaped) in yeast to convex (humped) in corn.

## Discussion

Mating preferences may diverge between populations as a result of genetic drift or because of divergent natural selection between environments [12]. Direct experimental tests of either of these mechanisms, however, are lacking. Here we used an experimental evolution approach, involving an ancestral laboratory and two novel resource environments, to evaluate the role of divergent selection in the evolution of female mating preferences among replicate populations of *D. serrata*. CHCs, the traits that predict male and female mating success, evolved in response to the new environments. Furthermore, as determined using more than 1,250 independent mate choice trials, female mating preferences for these same CHCs also diverged among populations, with a component of this divergence occurring consistently among treatment environments. This provides a direct experimental demonstration that mating preferences can evolve, at least in part, as a result of environmentally based divergent selection.

An important component of our experimental design was the choice of novel environments to which *D. serrata* populations were exposed. Mate choice in the *D. serrata* species complex is based largely on nonvolatile CHCs [31–37]. Divergent selection was applied using different resource environments, because CHC expression in insects depends on the amino acids available in their diet [44], and *Drosophila* CHCs are known to be affected by larval substrate [45]. It is therefore not surprising that these environments generated divergent natural selection on CHCs, nor that adaptation occurred in every CHC except 2-Me-C_26_, which alone did not display a significant univariate sex × treatment interaction (*p* = 0.420). However, male and female CHCs responded very differently to the novel environments. The response to selection of homologous traits will depend on the different selective optima for each sex and on the intersex genetic correlations [46]. Our results demonstrate that the level of sexual dimorphism in this species can evolve as predicted from the generally low intersex genetic correlations for these traits [32].

Under the assumption that the divergence of mating preferences among populations is a key source of premating isolation, our results are consistent with the classic by-product model of allopatric speciation. In this model, new species arise as a side effect of natural selection adapting populations to their different environments [12,23–25]. Although premating isolation has been shown to evolve in parallel in correlation with environment [12,23,26–30], little is known about the role of mating preferences in this process. Here we have used a direct experimental manipulation to show that mating preferences, a trait long thought to be an important source of premating isolation, can evolve, at least in part, in correlation with environment. Linear sexual selection exerted by female mating preferences diverged in the novel environments primarily on two male CHCs: 2-Me-C_26_ and 2-Me-C_28_. Correlational sexual selection exerted by female preferences between these two CHCs and a third, (Z)-9-C_25:1_, was also changed by selection in the novel environments.

There are two classes of mechanisms by which divergent selection between environments could cause consistent mating preference evolution. In the first, CHCs evolve by divergent natural selection, adapting to the different treatment environments. Mating preferences evolve as a correlated response to this, due to either pleiotropy or linkage disequilibrium between the genes involved in adaptation and those affecting mating preferences [19,21]. In the second, mating preferences diverge between environments either due to direct natural selection on the preferences themselves [20,47] or as a plastic response to the different environments. This preference divergence generates divergent sexual selection on signal traits (CHCs), and subsequent CHC divergence may feed back on preferences, furthering their divergence as a correlated response. Determining the mechanism responsible for preference divergence among environments in our populations will thus require additional information about the independent and combined roles of natural and sexual selection in the evolution of CHCs and mating preferences. This will require further experiments that independently manipulate the presence and absence of both forms of selection within a single experimental design [38].

Surprisingly, there was no strong association in our experiment between the CHCs that adapted and those involved in preference changes. Linear preferences changed most among environments for 2-Me-C_26_. However, as noted earlier, 2-Me-C_26_ is the only CHC for which there was no evidence of adaptation. Nonlinear preferences changed for correlational selection between CHCs (Z)-9-C_25:1_ and 2-Me-C_26_, and (Z)-9-C_25:1_ and 2-Me-C_28_. Both (Z)-9-C_25:1_ and 2-Me-C_28_ do show strong evidence of adaptation, although primarily in females. That adaptation of CHCs occurred to a greater extent in females than in males may seem surprising, given that models of sexual selection predict the coevolution of signal traits and preferences [13]. This could arise, however, if male CHCs were evolving primarily by sexual selection; it may take time for divergent female preferences to become established and to subsequently affect male trait evolution. Alternatively, if CHCs are condition-dependent and males allocate resources preferentially to them, CHCs themselves might evolve less than other traits competing for the available resources. Whatever the reason, the lack of an association between the evolution of male signal traits and female preference is a clear evolutionary outcome of our experiment and suggests that the evolution of signal traits and preferences in novel environments may be a more complex process than is currently appreciated.

All else being equal, the consistent evolution of mating preferences in correlation with environment should eventually cause the parallel evolution of premating isolation [12,26–30]. In our experiment, however, genetic drift was almost certainly responsible for a component of the among-population divergence in mating preferences, and this may ultimately counter the parallel evolution of preferences and premating isolation. Because we have only a single estimate of the linear and nonlinear mating preferences (sexual selection gradients) for each population, variation among populations within treatments is a consequence of both genetic drift and measurement error associated with each selection gradient. It is therefore not possible to distinguish between drift and measurement error in contributing to preference divergence among the populations in our experiment. Quantifying the importance of genetic drift in future experiments will be difficult because it requires the repeated measurement of population-level selection gradients.

Finally, changes in population-level mate preferences (i.e., sexual selection gradients) are ultimately the product of evolutionary changes in individual preference functions and/or their frequency within populations [48]. A complete understanding of how mating preferences diverge will thus also require additional studies that explore how natural selection and genetic drift affect the evolution of individual preferences. Knowledge of the underlying evolution in individual preferences responsible for changes in population-level mating preferences may have important implications for our understanding of speciation, because the shape and diversity of individual preference functions within populations will affect the evolution of reproductive isolation between populations. Determining how preference evolution generates reproductive isolation remains a central and untested issue in speciation research.

## Materials and Methods

### Experimental populations

In April of 2002, 12 populations of *D. serrata* were independently derived from the Forster stock previously described [31,32,37]. These populations were assigned to one of three treatment environments (yeast, rice, or corn), yielding four replicate populations in each environment. The environments varied in the food medium on which the flies were raised. The yeast treatment, representing the ancestral environment, used the same food medium that has been used to maintain this stock since its establishment in the laboratory in January, 1998. This is a standard laboratory medium (Table S2). In the novel environments, the majority of the yeast was replaced with either rice flour (rice environment) or corn starch (corn environment; Table S2). These two new environments were chosen because the expression of CHCs in insects is dependent on the amino acids available [44], and *Drosophila* CHCs are known to be affected by larval substrate [45]; different substrates have also been shown to affect the strength of reproductive isolation in another species *(D. mojavensis)* [49,50].

Populations were maintained with nonoverlapping generations by transferring approximately 100 individuals of unknown sex into each of two new bottles every generation. In October 2003, prior to measuring their CHCs and mating preferences, all flies from every population were raised on the ancestral yeast medium for two generations to remove any environmental effects. At this time, the populations on yeast were 37 generations old. Because generation times were slower in the novel environments, the corn and rice populations had each evolved for 29 generations.

### Measurements of CHCs

CHCs were extracted from individual flies as previously described [36]. Samples were analyzed using gas chromatography (GC) and flame ionization detection on an Agilent Technologies (Wilmington, Delaware, United States) 6890N gas chromatograph fitted with a HP5 column of 50 m × 0.32 mm internal diameter and a pulsed splitless front inlet. The temperature program began by holding at 57 °C for 1.1 min, then increased to 190 °C at 100 °C min^−1^, followed by 190–310 °C at 25 °C min^−1^, then finally holding at 310 °C for 5 min. Individual CHC profiles were determined by integration of the area under nine peaks. These are the same peaks as used in past studies [31–35], identified in order of their retention times as: (Z,Z)-5,9-C_24:2_; (Z,Z)-5,9-C_25:2_; (Z)-9-C_25:1_; (Z)-9-C_26:1_; 2-Me-C_26_; (Z,Z)-5,9-C_27:2_; 2-Me-C_28_; (Z,Z)-5,9-C_29:2_; and 2-Me-C_30_ [51].

Relative amounts of each of these nine CHCs were calculated for each individual by dividing the area under each peak by the total area under all their peaks. In GC analysis, this use of proportional peak areas is favored over absolute values because, even with the use of internal size standards, absolute values are often subject to large experimental error [36,52]. The use of proportions introduces nonindependence among peaks within the CHC profiles of individuals, because the area under any one peak influences the total area and thus the proportional values of other peaks. This unit-sum constraint, characteristic of compositional data, is removed using a logcontrast transformation [36,53]. Logcontrasts were calculated using the proportional area of (Z,Z)-5,9-C_24:2_ as the divisor, yielding eight logcontrast peak values for every individual. Because logcontrast peak values derive ultimately from proportional data, all analyses described below address changes in the relative abundance of CHCs within individuals. Statistical analyses were performed using SAS v. 8.2 (SAS Institute, Cary, North Carolina, United States).

### Adaptation of male and female CHCs

Ten virgin males and ten virgin females were collected from each population at eclosion using light CO_2_ anesthesia. Flies were held separately by sex in vials of five flies/vial; males were transferred singly into new vials after 2 d. All holding vials included a small amount of live yeast sprinkled on top of the food. At 4 d post-eclosion, CHC profiles of all flies were determined using GC as described above.

Differences in the CHC profiles of males and females from each population were tested using the following linear model:



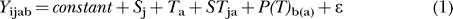



where *Y* is the logcontrast peak value for the *i*th CHC (*i* = 1 − 8), *S*_j_ is the effect of sex, *T*_a_ is the effect of treatment environment (yeast, rice, or corn), *ST*_ja_ is the effect of the interaction of sex with treatment, *P(T)*_b(a)_ is the effect of replicate populations nested within treatment, and *ɛ* is the error. Population was modeled as a random effect whereas all other factors were fixed. MANOVA was used to test the significance of all terms in this model. Because of the nested design, significance of the treatment effect was tested over the mean square (MS) of *P(T)*. All other terms were tested over MS_error_.

This model yielded a significant sex × treatment interaction, so to isolate the combination of CHCs that were responsible for this response to selection, we conducted a multifactorial canonical discriminant analysis. This entailed performing an eigenanalysis on the resultant matrix of E^−1^H, where E was the sums of squares and cross-product (SSCP) matrix of the *ɛ*_ijk_ term, and H was the SSCP hypothesis matrix for the sex × treatment interaction [54]. The first two eigenvectors (CVs) accounted for 100% of the variance at the sex × treatment level.

### Measurement of female preferences for male CHCs

Female mating preferences can be measured in two fundamentally different ways [48]. First, preference functions can be determined for individual females [4]. While this approach allows the diversity of female preferences within populations to be visualized, statistical descriptions of these functions can be complex, making comparisons among populations (our goal here) difficult. Alternatively, population-level sexual selection gradients [55] may be used to describe the form and strength of sexual selection (female preference) operating on male traits. We use this approach because comparison of sexual selection gradients among populations is straightforward to accomplish within a well-described modeling framework [56].

Female choice trials were conducted in glass vials containing 8 ml of standard yeast medium and using 4-d-old virgin flies that had been held as described above. In each trial, a single female from one of the experimental populations was placed together with two 4-d-old virgin males from the ancestral laboratory population (Forster). These males were used to provide a standard for comparison of female preferences among populations; their use means that any differences among populations can be attributed unambiguously to the evolution of female preference as opposed to the males from which the females are choosing. Forster males were raised and held using the same protocol as described above for the experimental populations.

An average of 106 trials (range 96–112) were conducted for each of the 12 populations, and mating occurred in nearly all (> 99%) of them. Mating vials were observed, and once intromission had been achieved between the female and one of the two males, all flies were anesthetized with CO_2_ and the chosen and rejected males had their CHCs extracted for subsequent GC analysis (females were discarded). CHC profiles of each male were integrated and proportional peak areas were logcontrast transformed.

### Characterizing sexual selection within populations

Linear sexual selection on the eight male logcontrast CHCs arising from female mating preferences was analyzed separately in each population using the standard first-order polynomial regression model [55]. Although male mating success was binomially distributed in these analyses, parametric significance testing was performed in all cases using standard methods within a linear model framework, because when sample sizes are large and the probability of either outcome is equal (as in the present case), the binomial distribution provides an excellent approximation of the normal distribution. Results of bootstrap analyses from past experiments have confirmed the accuracy of this approximation [32]. Males were treated as independent replicates in these analyses; this has no discernable effect on the significance of individual selection gradients when compared with treating females as replicates (Figure S1 and S2; Protocol S1).

To confirm the presence of sexual selection on male CHCs by female mating preferences in our populations, we estimated, separately for each population, the strength of linear sexual selection on the eight logcontrast CHCs for each male. Similar to past studies [31–35], multicollinearity among these logcontrast CHCs was minimal, so these values were used directly in the analysis. From these regressions, the proportion of total variation in male mating success accounted for by linear sexual selection on all eight logcontrast CHCs was given by the adjusted coefficient of determination (*r* ^2^), with significance indicated by the overall fit of the model.

To evaluate the overall significance of nonlinear selection on the eight logcontrast CHCs in our populations, we first conducted a canonical rotation to place all nonlinear selection on the eight eigenvectors of the matrix of quadratic and cross-product terms (i.e., the gamma matrix) in each population, thus eliminating all cross-product terms [57]. Nonlinear sexual selection on these eight eigenvectors was then analyzed using the standard second-order polynomial regression model [55,58]. The overall significance of all nonlinear selection in each population was then evaluated using partial *F*-tests [35,59] that compared the fit of the models with and without the eight quadratic terms. Conducting such a rotation does not affect the amount of nonlinear selection present in each population, but does increase the likelihood of detecting its significance by reducing the number of nonlinear coefficients from 36 to eight.

### Variation among populations in sexual selection

To determine if female mating preferences diverged among populations, we tested for variation among populations in both linear and nonlinear sexual selection on male CHCs. Among-population variation in linear sexual selection was tested using the following model:



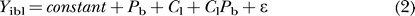



where *Y* is the mating success of the *i*th male from the *b*th population (*b* = 1 − 12). *C*_l_ is the effect on male mating success of the *l*th male logcontrast CHC, representing linear sexual selection on this trait. Variation among populations in linear sexual selection on male logcontrast CHC peak value *l* would be indicated by a significant *C*_l_ *P*_b_ interaction. To evaluate whether linear sexual selection varied among the twelve populations, we used a single partial *F*-test [35,59] that compared the fit of the above model with one lacking all of the *C*_l_ *P*_b_ interactions.

Among-population variation in nonlinear sexual selection was evaluated in an analogous manner using the following model:



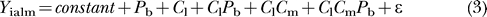



where *C*_l_ *C*_m_ is the combined effect on male mating success of the *l*th and *m*th male logcontrast CHCs, representing nonlinear selection (quadratic: *l* = *m*; correlational: *l* ≠ *m*) on these traits. Variation among populations in nonlinear sexual selection would be indicated by a significant *C*_l_ *C*_m_ *P*_b_ interaction. To evaluate whether nonlinear selection varied among populations overall, we again used a single partial *F*-test [35,59] that compared the fit of this model with one lacking all of the *C*_l_ *C*_m_ *P*_b_ interactions. By excluding from the reduced model the interactions of all nonlinear terms with population, significance of this partial *F*-test reflects the combined importance of among-population variation in all forms of nonlinear sexual selection.

### Variation among treatments in sexual selection

To determine if natural selection adapting populations to their novel treatment environments caused consistent mating preference divergence, we tested for variation among treatments in both linear and nonlinear sexual selection on male CHCs. Among-treatment variation in linear sexual selection was tested using the following model:



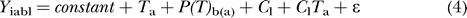



where the *b*th population is nested within the *a*th treatment environment (yeast, rice, and corn). Treatment was modeled as a fixed effect and population was modeled as a random effect nested within treatment. Variation among treatments in linear sexual selection on male logcontrast CHC peak value *l* would be indicated by a significant *C*_l_ *T*_a_ interaction. To evaluate whether linear sexual selection varied among treatments overall, we used a single partial *F*-test [35,59] that compared the fit of the above model with one lacking all of the *C*_l_ *T*_a_ interactions.

Among-treatment variation in nonlinear sexual selection was evaluated in a similar manner using the following model:



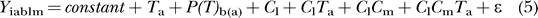



Variation among treatments in nonlinear sexual selection would be indicated by a significant *C*_l_ *C*_m_ *T*_a_ interaction in this model. To evaluate whether nonlinear selection varied among treatments overall, we again used a single partial *F*-test [35,59] that compared the fit of this model with one lacking all of the *C*_l_ *C*_m_ *T*_a_ interactions.

The contribution of divergent selection to the among-population diversification of mating preferences was quantified using MS ratios. For linear sexual selection, the total among-population variation is represented by the MS (*C*_l_ *P*_b_) from Equation 2, and the among-treatment variation by the MS (*C*_l_ *T*_a_) from Equation 4. Their ratio, MS (*C*_1_ *T*_a_) / MS (*C*_l_ *P*_b_), is an estimate of the proportion of the total among-population variation in mating preferences that is grouped by treatment environment. For nonlinear sexual selection, this ratio is MS (*C*_l_ *C*_m_ *T*_a_) / MS (*C*_l_ *C*_m_ *P*_b_), obtained from Equations 5 and 3, respectively, and is an estimate of the proportion of the total among-population variation in nonlinear mating preferences that is grouped by treatment environment.

## Supporting Information

Figure S1 Quantifying Potential Bias in the Magnitude of Selection Gradients Caused by Pseudoreplication Mean sexual selection gradients on eight logcontrast male CHCs from three geographic populations are presented. For each population, mean selection gradients were estimated from 1,000 bootstrap replicates each of two subpopulations composed of 128 males (64 chosen, 64 rejected) randomly sampled from the population of 256 males. In subpopulation A, 64 trials were randomly selected, and both the chosen and rejected males were used. In subpopulation B, a single male (chosen or rejected) was sampled from each of the 128 trials. The line is a one-to-one line.
(19 KB CDR).

Figure S2 Quantifying Potential Bias in the Significance Level of Selection Gradients Caused by Pseudoreplication Mean significance levels for the sexual selection gradients on eight logcontrast male CHCs from three geographic populations estimated from 1,000 bootstrap replicates of subpopulations A and B as described in Figure S1. The line is a one-to-one line.
(19 KB CDR).

Table S1 Standardized Linear and Nonlinear Sexual Selection Gradients on the Eight Male CHCs for Each of the 12 Populations (45 KB WPD).

Table S2 Media Recipes for the Three Treatment Environments (19 KB WPD).

Protocol S1 Pseudoreplication and Multiple-Choice Mating Trials (24 KB WPD).

## Abbreviations

CHC: cuticular hydrocarbon
CV: canonical variate
GC: gas chromatography
MS: mean square
