# Correction: Divergent Selection and the Evolution of Signal Traits and Mating Preferences

**DOI:** 10.1371/journal.pbio.1001836

**Published:** 2014-03-17

**Authors:** The PLOS Biology Staff

The Supporting Figures are currently provided as CDR files and the Supporting Tables and Protocol are provided as WPD documents. For any readers unable to access these files types, the authors have provided PDF files of all Supporting Information files.

## Supporting Information

Figure S1 **Quantifying Potential Bias in the Magnitude of Selection Gradients Caused by Pseudoreplication.** Mean sexual selection gradients on eight logcontrast male CHCs from three geographic populations are presented. For each population, mean selection gradients were estimated from 1,000 bootstrap replicates each of two subpopulations composed of 128 males (64 chosen, 64 rejected) randomly sampled from the population of 256 males. In subpopulation A, 64 trials were randomly selected, and both the chosen and rejected males were used. In subpopulation B, a single male (chosen or rejected) was sampled from each of the 128 trials. The line is a one-to-one line.
doi:10.1371/journal.pbio.0030368.sg001

Figure S2 **Quantifying Potential Bias in the Significance Level of Selection Gradients Caused by Pseudoreplication.** Mean significance levels for the sexual selection gradients on eight logcontrast male CHCs from three geographic populations estimated from 1,000 bootstrap replicates of subpopulations A and B as described in Figure S1. The line is a one-to-one line.
doi:10.1371/journal.pbio.0030368.sg002

Table S1 **Standardized Linear and Nonlinear Sexual Selection Gradients on the Eight Male CHCs for Each of the 12 Populations.**
doi:10.1371/journal.pbio.0030368.st001

Table S2 **Media Recipes for the Three Treatment Environments.**
doi:10.1371/journal.pbio.0030368.st002

Protocol S1 **Pseudoreplication and Multiple-Choice Mating Trials.**
doi:10.1371/journal.pbio.0030368.sd001
