# Supplementary figures and images for: Correction: Divergent Selection and the Evolution of Signal Traits and Mating Preferences
Source: PLoS Biol. 2014 Mar 17;12(3):e1001836. doi: 10.1371/journal.pbio.1001836 (PMC3956494; doi:10.1371/journal.pbio.1001836)

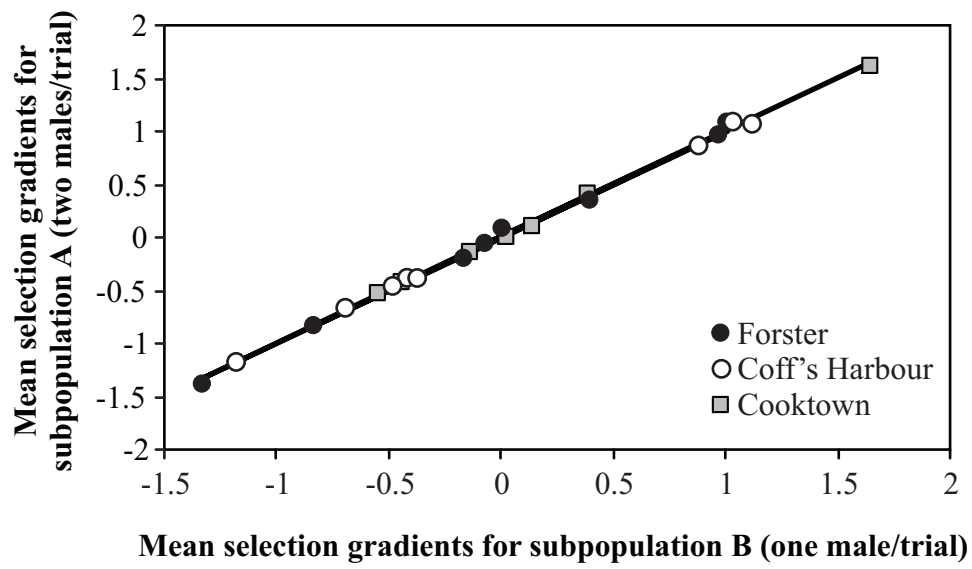

Supplement: Figure S1 — Quantifying Potential Bias in the Magnitude of Selection Gradients Caused by Pseudoreplication. Mean sexual selection gradients on eight logcontrast male CHCs from three geographic populations are presented. For each population, mean selection gradients were estimated from 1,000 bootstrap replicates each of two subpopulations composed of 128 males (64 chosen, 64 rejected) randomly sampled from the population of 256 males. In subpopulation A, 64 trials were randomly selected, and both the chosen and rejected males were used. In subpopulation B, a single male (chosen or rejected) was sampled from each of the 128 trials. The line is a one-to-one line. doi:10.1371/journal.pbio.0030368.sg001 [file pbio.1001836.s001.pdf]

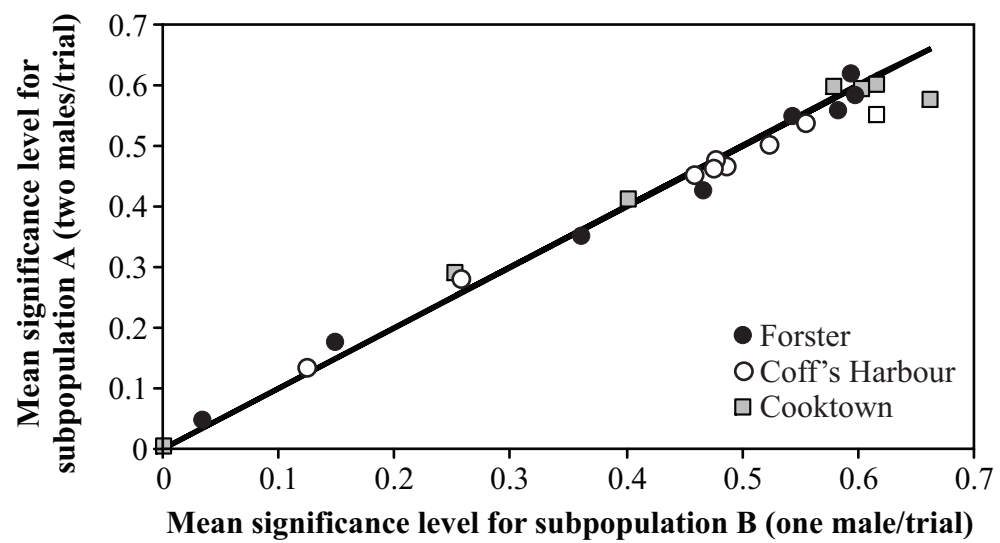

Supplement: Figure S2 — Quantifying Potential Bias in the Significance Level of Selection Gradients Caused by Pseudoreplication. Mean significance levels for the sexual selection gradients on eight logcontrast male CHCs from three geographic populations estimated from 1,000 bootstrap replicates of subpopulations A and B as described in Figure S1. The line is a one-to-one line. doi:10.1371/journal.pbio.0030368.sg002 [file pbio.1001836.s002.pdf]
