# Supplementary material for: Correction: Divergent Selection and the Evolution of Signal Traits and Mating Preferences
Source: PLoS Biol. 2014 Mar 17;12(3):e1001836. doi: 10.1371/journal.pbio.1001836 (PMC3956494; doi:10.1371/journal.pbio.1001836)
Supplement: Table S1 — Standardized Linear and Nonlinear Sexual Selection Gradients on the Eight Male CHCs for Each of the 12 Populations. doi:10.1371/journal.pbio.0030368.st001 [file pbio.1001836.s003.pdf]

Table S1. Vector of standardized directional selection gradients (β) and the matrix (γ) of standardized quadratic and correlational selection gradients indicating the strength of sexual selection on eight male logcontrast CHCs for each of the four replicate populations from the ancestral yeast (A) and the novel rice (B) and corn (C) treatment environments. For each population, the sexual selection gradients are the partial regression coefficients of the eight standardized male logcontrast CHCs on relative mating success. Linear and nonlinear gradients were estimated in separate regressions. For each gradient, the four replicate populations are listed in order (one to four) within each treatment environment.

A) Yeast

		γ							
	β	Z,Z-5,9-$C_{25:2}$	Z-9-$C_{25:1}$	Z-9-$C_{26:1}$	2-Me-C_{26}	Z,Z-5,9-$C_{27:2}$	2-Me-C_{28}	Z,Z-5,9-$C_{29:2}$	2-Me-C_{30}
Z,Z-5,9-$C_{25:2}$	-0.154*	-0.088	-0.052	-0.027	-0.396*	0.050	0.507*	0.115	-0.239*
	-0.018	0.029	-0.087	0.015	0.048	0.010	-0.188	0.096	-0.052
	-0.037	0.042	-0.117	-0.107	-0.159	0.091	0.079	0.063	-0.133
	-0.016	-0.084	0.215	0.116	0.100	0.187	-0.175	-0.045	0.035
Z-9-$C_{25:1}$	-0.109		0.017	0.106	0.326*	-0.108	-0.147	0.079	0.110
	-0.042		0.058	-0.048	-0.134	0.012	0.233	-0.046	-0.013
	-0.097		0.029	-0.023	-0.085	-0.129	0.156	0.072	-0.065
	-0.112		-0.066	-0.139	-0.250*	-0.022	0.167	0.035	-0.125
Z-9-$C_{26:1}$	-0.029			-0.113*	0.006	0.119	-0.006	-0.009	-0.034
	-0.023			0.042	0.128	-0.061	-0.124	0.048	0.050
	-0.011			0.105*	-0.105	0.043	0.146	-0.093	-0.084
	-0.085			-0.043	-0.027	-0.055	0.057	0.031	-0.082
2-Me-C_{26}	0.209*				0.038	0.047	-0.058	-0.116	0.181
	0.164				0.056	0.053	-0.202	-0.123	-0.157
	0.043				0.182	0.049	-0.271	0.128	0.045
	0.080				0.028	-0.015	-0.054	0.026	0.047
Z,Z-5,9-$C_{27:2}$	-0.078					-0.031	-0.109	0.044	0.169
	-0.165*					-0.118	0.074	0.165	-0.141
	-0.080					-0.114	-0.103	0.047	0.200
	-0.113					-0.162	0.234	0.228	-0.128
2-Me-C_{28}	-0.100						0.037	-0.001	-0.157
	-0.088						0.145	0.030	0.260
	0.245*						0.093	-0.012	-0.098
	0.182						-0.103	-0.054	0.124
Z,Z-5,9-$C_{29:2}$	0.711*							-0.145*	0.052
	0.779*							-0.227*	0.059
	0.581*							-0.137*	-0.034
	0.617*							-0.207*	0.059
2-Me-C_{30}	-0.348*								0.004
	-0.254*								-0.074
	-0.430*								-0.025
	-0.428*								-0.076

B) Rice

	β	γ Z,Z-5,9-$C_{25:2}$	Z-9-$C_{25:1}$	Z-9-$C_{26:1}$	2-Me-C_{26}	Z,Z-5,9-$C_{27:2}$	2-Me-C_{28}	Z,Z-5,9-$C_{29:2}$	2-Me-C_{30}
Z,Z-5,9-$C_{25:2}$	0.057	-0.082	0.164	-0.118	-0.125	0.222	0.126	-0.090	-0.009
	-0.126	-0.049	0.028	0.028	0.195	0.078	-0.165	0.153*	0.042
	-0.119	-0.018	0.139	0.173*	-0.169	-0.361*	0.458*	-0.041	0.067
	-0.123	0.035	0.023	0.046	-0.18	-0.017	0.127	-0.082	-0.142
Z-9-$C_{25:1}$	-0.154*		-0.042	0.092	-0.043	-0.088	0.153	-0.001	-0.092
	0.035		0.024	-0.077	-0.180	-0.007	0.174	0.000	-0.050
	-0.073		0.071	-0.032	-0.030	-0.248*	0.076	0.123	0.031
	-0.073		-0.047	0.114	-0.123	-0.126	0.156	0.061	0.026
Z-9-$C_{26:1}$	-0.050			0.063	-0.117	0.052	0.139	-0.014	0.010
	-0.050			0.078	0.028	0.060	-0.073	0.008	-0.054
	0.001			0.023	-0.044	-0.142	0.123	0.065	-0.027
	-0.075			0.041	-0.054	0.014	-0.121	-0.074	0.003
2-Me-C_{26}	-0.046				-0.107	0.090	0.300	-0.218	0.023
	0.028				0.101	0.157	-0.413	-0.026	0.137
	-0.001				-0.026	0.156	0.120	-0.025	0.122
	0.009				-0.005	0.092	0.364	-0.185	-0.112
Z,Z-5,9-$C_{27:2}$	-0.158*					-0.200*	-0.002	0.131	0.000
	-0.203*					-0.026	-0.198	0.098	0.016
	-0.157*					0.193	-0.181	0.109	0.003
	-0.132					-0.118	0.057	0.293*	0.077
2-Me-C_{28}	0.167						-0.086	0.231	-0.095
	0.076						0.258	-0.050	-0.097
	0.252*						-0.163	-0.049	-0.093
	0.173						-0.361*	0.174	0.093
Z,Z-5,9-$C_{29:2}$	0.602*							-0.179*	0.113
	0.777*							-0.228*	0.068
	0.537*							-0.078	0.098
	0.715*							-0.266*	0.062
2-Me-C_{30}	-0.402*								0.032
	-0.238*								0.035
	-0.343*								-0.023
	-0.309*								-0.045

C) Corn

		γ							
	β	Z,Z-5,9-$C_{25:2}$	Z-9-$C_{25:1}$	Z-9-$C_{26:1}$	2-Me-C_{26}	Z,Z-5,9-$C_{27:2}$	2-Me-C_{28}	Z,Z-5,9-$C_{29:2}$	2-Me-C_{30}
	-0.130*	0.018	-0.034	0.024	-0.117	-0.103	0.223	0.124	-0.036
	-0.067	0.005	-0.001	-0.049	-0.091	0.057	0.064	0.043	-0.110
	-0.041	-0.072	0.017	0.084	0.091	0.098	-0.168	0.133	-0.060
Z,Z-5,9-$C_{25:2}$	-0.065	-0.109	0.055	0.036	-0.015	0.374*	-0.105	-0.043	0.010
	-0.008		-0.017	0.002	0.119	0.139	-0.157	0.088	-0.018
	-0.175*		-0.015	0.103	0.152	-0.030	-0.211*	0.078	0.004
	-0.148*		0.057	-0.066	0.063	-0.058	-0.189	0.074	0.156
Z-9-$C_{25:1}$	-0.080		0.015	-0.015	-0.032	-0.082	-0.065	0.079	-0.012
	0.062			0.025	0.206	-0.088	-0.155	-0.029	0.128
	-0.045			0.062	-0.165	-0.199*	0.090	0.200*	-0.009
	0.005			-0.017	0.169	0.035	-0.122	-0.036	-0.034
Z-9-$C_{26:1}$	0.000			0.044	0.067	-0.004	-0.091	0.047	-0.035
	-0.037				0.081	0.082	-0.235	0.059	-0.048
	0.080				0.114	0.143	-0.095	-0.042	0.322*
	0.218*				0.133	0.196	-0.082	-0.132	0.100
2-Me-C_{26}	-0.091				0.047	-0.142	-0.118	0.108	0.094
	-0.214*					0.067	-0.186	0.036	-0.110
	-0.171*					0.047	-0.064	0.075	0.123
	-0.092					-0.064	0.040	0.060	-0.002
Z,Z-5,9-$C_{27:2}$	-0.200*					-0.240*	0.257	0.143	-0.134
	0.263*						0.087	-0.080	0.149
	0.141						0.079	-0.008	-0.298*
	-0.039						-0.025	-0.015	0.052
2-Me-C_{28}	0.221*						0.102	-0.161	-0.128
	0.752*							-0.186*	0.071
	0.758*							-0.171*	0.131
	0.530*							-0.044	0.154
Z,Z-5,9-$C_{29:2}$	0.704*							-0.219*	0.220*
	-0.332*								-0.099
	-0.368*								0.080
	-0.410*								-0.061
2-Me-C_{30}	-0.438*								0.008
