# Supplementary material for: Correction: Divergent Selection and the Evolution of Signal Traits and Mating Preferences
Source: PLoS Biol. 2014 Mar 17;12(3):e1001836. doi: 10.1371/journal.pbio.1001836 (PMC3956494; doi:10.1371/journal.pbio.1001836)
Supplement: Table S2 — Media Recipes for the Three Treatment Environments. doi:10.1371/journal.pbio.0030368.st002 [file pbio.1001836.s004.pdf]

Table S2. Media recipes for the three treatment environments.

	Treatment environment		
Ingredient	yeast	rice	corn
water	1L	1L	1L
agar	19g	19g	19g
raw sugar	54g	54g	54g
inactivated torula yeast	36g	3.6g	9g
rice flour	0g	32.4g	0
corn starch	0g	0g	27g
nipagin*	12ml	12ml	12ml
proprionic acid	6ml	6ml	6ml

*nipagin is a 10% w/v solution of methyl-4-hydrobenzoate in methanol
