# Supplementary material for: Correction: Divergent Selection and the Evolution of Signal Traits and Mating Preferences
Source: PLoS Biol. 2014 Mar 17;12(3):e1001836. doi: 10.1371/journal.pbio.1001836 (PMC3956494; doi:10.1371/journal.pbio.1001836)
Supplement: Protocol S1 — Pseudoreplication and Multiple-Choice Mating Trials. doi:10.1371/journal.pbio.0030368.sd001 [file pbio.1001836.s005.pdf]

Text S1. When observations in an experimental design that share the same treatment application are mistakenly considered independent, pseudoreplication has occurred [1]. In multiple choice mating trials, separate males presented to a single females may be considered non-independent replicates because the independent application of the treatment (female choice) is the female herself. Since females represent the maximum number of independent observations, only a single male phenotype can then be associated with each female in the statistical analysis. However, because mate choice will always imply some dependence among observations on male phenotypes, under field or laboratory conditions, avoiding pseudoreplication when assessing mate choice may be particularly difficult in many circumstances, and the appropriate degrees of freedom to be used in the analysis deserves some consideration.

In the presence of pseudoreplication, the chance of incorrectly rejecting a null hypothesis of interest (type I error) increases as the experimental error associated with each application of the treatment is underestimated. In many experimental designs, the number of independent applications of the treatment are few and the number of observations within independent applications are large, resulting in a substantial increase in type I error [see Fig. 4 in ref. 56]. In the mate choice experimental design used here, however, the opposite is true; the number of independent applications of female choice (the number of females) is large, while the minimum possible number of dependent observations were used within each application (two male phenotypes). In such a case, if the number of independent applications of the treatment is sufficient to encompass all of the experimental variation (both biological and physical) that exists among females, the increase in type I error will be negligible.

To directly test for an increase in type I error that may have occurred by representing males as independent replicates, we analysed a data set that uses the same experimental design as the one employed here, but in which the chosen and rejected males from each female could be identified (M. Higgie, unpublished data). Using this data, sexual selection gradients were estimated for three separate geographical populations of *D. serrata* (Cooktown, Forster and Coffs Harbour) in an identical fashion to the methods used in the current manuscript. The Forster population is the same laboratory stock from which the experimental populations in the current manuscript were derived; the use of the other two populations will give some indication of how variation among populations in female preference might affect the level of pseudoreplication generated.

Because chosen and rejected males from individual females were given unique identifiers in this data set, we can determine if the non-independence of pairs of males presented to a single female causes an increase in type I error. This was done by conducting a bootstrap resampling analysis (with replacement) of two subpopulations of the data for each of the three geographic populations. First, selection gradients were calculated using pairs of males from each mate choice trial (chosen and rejected) as independent replicates, but restricting the number of trials (females) to half the total number conducted (subpopulation A). Second, selection gradients were calculated using only a single male from each mate choice trial, with a male (chosen or rejected) being sampled randomly from each trial under the condition that the total number of chosen and rejected individuals was equal (subpopulation B). In this fashion, the same total number of males (128), and the same proportion of chosen and rejected males (50:50), are present in each subpopulation; the only difference between the two being that the number of female trials in subpopulation A is half that in subpopulation B (64 vs. 128). 1000 bootstrapped replicates each

of subpopulation A and B were then generated and the mean magnitude and significance levels of each of the eight estimated sexual selection gradients on male logcontrast CHC values were calculated for each geographic population.

From this analysis, there is no indication that using both males from a trial causes any bias in the magnitude of the sexual selection gradients. The mean selection gradients estimated using the two techniques (i.e. subpopulations A and B) are highly concordant, clustering tightly along the one-to-one line for all three geographical populations (Fig. S1). There is also no indication that using both males from a trial is of any concern with regards to the significance of these selection gradients. An increase in type I error caused by pseudoreplication is primarily of concern for smaller P-values because it is changes in these that can bias conclusions regarding the significance of experimental treatments. In our data sets, however, there is excellent correspondence in P-values between the two techniques and, most importantly, there is no evidence of an increase in type I error for the smaller P-values (Fig. S2). In fact, for all six selection gradients with P-values less than 0.3 (which includes two gradients from each of the three geographical populations), the values are slightly conservative when both males are used from each mate choice trial (points fall above the one-to-one line). This indicates that for all three populations, including the Forster laboratory stock that is the ancestor of our current 12 experimental populations, sample sizes of 65 females are sufficient to capture the majority of variation among females. Because sample sizes in our current experiment were larger than those in this analysis, averaging 106 (range 96-112) females for each of the twelve populations, we conclude that non-independence of males is not a concern here.

References

1. Hurlbert SH (1984) Pseudoreplication and the design of ecological field experiments. Ecol Monogr 54: 187-211.
